# Variation in the proportion of the segregating genome shared between full-sibling cattle and sheep

**DOI:** 10.1186/s12711-023-00802-5

**Published:** 2023-04-18

**Authors:** David Kenny, Donagh P. Berry, Thierry Pabiou, Pierce Rafter

**Affiliations:** 1 Animal and Grassland Research and Innovation Centre, Teagasc, Moorepark, Fermoy, Ireland; 2 Sheep Ireland, Highfield House, Shinagh, Bandon, Ireland; 3 Irish Cattle Breeding Federation, Highfield House, Shinagh, Bandon, Ireland

## Abstract

The construction of covariance matrices that account for the genetic relationships among individuals, using pedigree or genotype data, is integral to genetic evaluations, which are now routinely used in the field of animal breeding. The objective of the present study was to estimate the standard deviation in the proportion of the segregating genome that is shared between pairs of full-sibling cattle and sheep independently. Post edits, genotype data comprising 46,069 autosomal single nucleotide polymorphisms (SNPs) were available for 4532 unique full-sibling sheep pairs, as well as for their respective parents. Post edits, genotypes from 50,493 autosomal SNPs were also available for 10,000 unique full-sibling cattle pairs, as well as their respective parents. Genomic relationship matrices were constructed for the sheep and cattle populations, separately. After accounting for both parental genomic inbreeding and the genomic relationship between both parents, the standard deviation in full-sibling cattle and sheep genomic relationships was 0.040 and 0.037 units, respectively. In addition, the intercept value from a linear regression model which regressed each full-sibling genomic relationship on both sire and dam inbreeding, as well as the genomic relationship between the parents, was 0.499 (0.001) for sheep and 0.500 (0.001) for cattle, conforming to the expectation that full-siblings, on average, share 50% of their segregating genome.

**Supplementary Information:**

The online version contains supplementary material available at 10.1186/s12711-023-00802-5.

## Background

The construction of covariance matrices that account for the genetic relationships between individuals is integral to the estimation of the genetic merit of animals [1, 2]. This is on the basis that such matrices, when included in genetic evaluations, partition the phenotypic variance in the observed trait into the causal variance due to genetic factors and the variance due non-genetic factors, including environmental factors [1, 2]. Traditionally, these covariance matrices used ancestry (i.e., pedigree) information to determine the expected relationships among animals [3]. The availability of genotype information has, in recent years, provided a means of deriving, more precisely, the actual extent to which two animals share their genome, as well as the extent of inbreeding in the animal itself [4]. Previously based on theory, Hill [5] estimated that the standard deviation in the actual genetic relationships between human full-siblings, in the absence of inbreeding, was 3.9 percentage units. Furthermore, using real data, Wang et al. [6] estimated that the standard deviation in the difference between genomic- and pedigree-based estimates of genetic relationships between full- and half-sibling chickens was 4.0 percentage units. In a study similar to that of Wang et al. [6], Pryce et al. [7] reported that the correlation between the genomic relationship and the pedigree-based estimate of relationships in cattle was 0.87 when eight generations of ancestry data were available for each animal in the dataset. If the available pedigree information is both deep and accurate, the deviation in actual relationships based on genotype information from expected relationships based on pedigree information can largely be attributed to Mendelian sampling [6]. Selective breeding acts on segregating genetic variants within a population with the aim to increase the frequency of alleles that additively contribute to (a) trait(s) of interest. Genetic evaluations use genetic relationship matrices as an estimate of the actual proportion of the segregating genome shared between animals to estimate the genetic merit of individual animals. The greater accuracy of the genome-based estimate of the actual genetic relationship over the pedigree-based estimate is the basis for the improvement in accuracy associated with breeding values estimated via genomic best linear unbiased prediction [4]. On this basis, knowledge of the extent of the variation in genomic relationships between individuals with similar expected pedigree relationships is of interest. Knowledge of the variation in genomic relationships among individuals relative to the expectation may be of interest when deciding whether to estimate the actual genetic relationship between animals using ancestry records or genotype information. In spite of this, the extent of the variability in the proportion of the segregating genome shared between full-siblings due to Mendelian sampling has, to the best of our knowledge, yet to be reported in both cattle and sheep. Therefore, the aim of the current study was to estimate the standard deviation in the proportion of the segregating genome shared between full-siblings due to Mendelian sampling in separate cattle and sheep populations, using genotypes from the full-siblings and their respective parents. In the present analysis, only full-siblings were considered to ensure that the dataset was free of any bias due to varying pedigree depth or an incorrect assumption of unrelatedness between founder animals.

## Methods

### Genetic datasets

Single nucleotide polymorphism (SNP) genotypes were obtained from Sheep Ireland for 32,549 sheep genotyped on the Ovine SNP50 genotype array (54,241 SNPs; Illumina Inc. San Diego, CA). Much of the sheep population consisted of purebred Belclare, Charollais, Suffolk, Texel, Vendeen, or a crossbred animal with a breed composition that was a combination of those five sheep breeds. This was complemented by SNP data on approximately 50,000 SNPs (depending on the genotype panel used) for 34,308 cattle. The cattle population comprised both purebred and crossbred cattle, of which the majority were purebred or a crossbred combination of the following breeds: Aubrac, Angus, Belgium Blue, Blonde d’Aquitaine, Charolais, Hereford, Holstein–Friesian, Jersey, Limousin, Salers, Shorthorn, and Simmental. All genotyped animals had a call rate higher than 95%. Single nucleotide polymorphisms on the X, Y, and mitochondrial chromosomes, and those that had a call-rate lower than 95% and/or deviated from Mendelian inheritance in more than 2% of the parent-progeny trios were removed. After editing, 46,069 SNPs were available for all genotyped sheep and 50,493 SNPs were available for all genotyped cattle. The cattle and sheep populations both consisted entirely of genotyped sires and dams with at least two genotyped full-sibling offspring. The sheep population comprised 407 sires, 2410 dams, and 6647 offspring and the cattle population comprised 5959 sires, 9091 dams and 19,258 offspring. The familial relationships between offspring, sire and dam were determined using pedigree records obtained from the Irish national sheep database and cattle database curated by Sheep Ireland and the Irish Cattle Breeding Federation, respectively. All full-sibling pairs and their respective parents were verified with the available genotype information. Monozygotic twins were removed.

Sporadically missing genotypes were imputed in the sheep population using the entire sheep dataset and the FImpute software suite [8]. The imputation pipeline for the cattle genotypes was also carried out using the FImpute software suite [8], with the pipeline imputing sporadically missing genotypes in cattle to a panel density of 52,455 autosomal SNPs. The imputation in cattle included a multi-breed reference population of 8650 males and 41,350 females selected based on their contribution to the Irish cattle population.

### Statistical analysis of the genetic relationships

A genomic relationship matrix (GRM) was constructed separately for the sheep and cattle populations using all available genotype data, following VanRaden's method 1 [9]:

1 $${\mathbf{GRM}}{\text{ = }}\frac{{{\mathbf{zz}}^{\prime } }}{{2\sum {p_{i} \left( {1 - p_{i} } \right)} }},$$

where ${p}_{i}$ is the frequency of the second allele at locus $i$, and $\mathbf{Z}$ is a matrix of centered genotype markers derived by subtracting the ${i}^{th}$ column of $\mathbf{M}$, the matrix of genotype markers which were coded as −1, 0 and 1 for the homozygous, heterozygous and opposing homozygous genotypes, respectively, at locus $i$, by ${2(p}_{i}-0.5)$ such that each column of $\mathbf{Z}$ has a mean of 0.

The genomic relationship between full-siblings was modelled separately in sheep and cattle using the following ordinary least squares regression model:

2 $$\tt {\text{SibR}} = \upmu + \upalpha {\text{ParR}} + \upbeta {\textit{F}}_{{sire}} + \upgamma {\textit{F}}_{{dam}} + e,$$

where $\mathrm{SibR}$ is the genomic relationships between the full-sibs, μ is the intercept, $\mathrm{ParR}$ is the genomic relationships between the sires and the dams of the full-siblings and $\upalpha$ is the associated regression coefficient, ${F}_{\mathrm{sire}}$ is the genomic inbreeding coefficients of the sires (i.e., diagonal element of the GRM for the sire minus 1), $\upbeta$ is the regression coefficient associated with sire inbreeding, ${F}_{\mathrm{dam}}$ is the genomic inbreeding coefficients of the dams (i.e., diagonal element of the GRM for the dam minus 1), $\upgamma$ is the regression coefficient for dam inbreeding, and $\mathrm{e}$ is the residual term for the model. The proportion of the variance in the genomic relationship between full-siblings accounted for by the model was evaluated using the R^2^ statistic [10].

The expected variance in the proportion of the segregating genome shared between full-siblings was calculated using the formula derived by Visscher [11, 12] and further developed by Hill [5]:

3 $${\sigma }_{R}^{2}=\frac{1}{16L}-\frac{1}{3{L}^{2}},$$

where $L$ is the length of the genome in Morgan. Visscher’s formula was used to calculate the approximate variance in the genomic relationship between full-siblings for cattle based on a genome length of 32.5 Morgan [5] and again separately for sheep using a genome length of 31.9 Morgan [13].

## Results

**Fig. 1:**
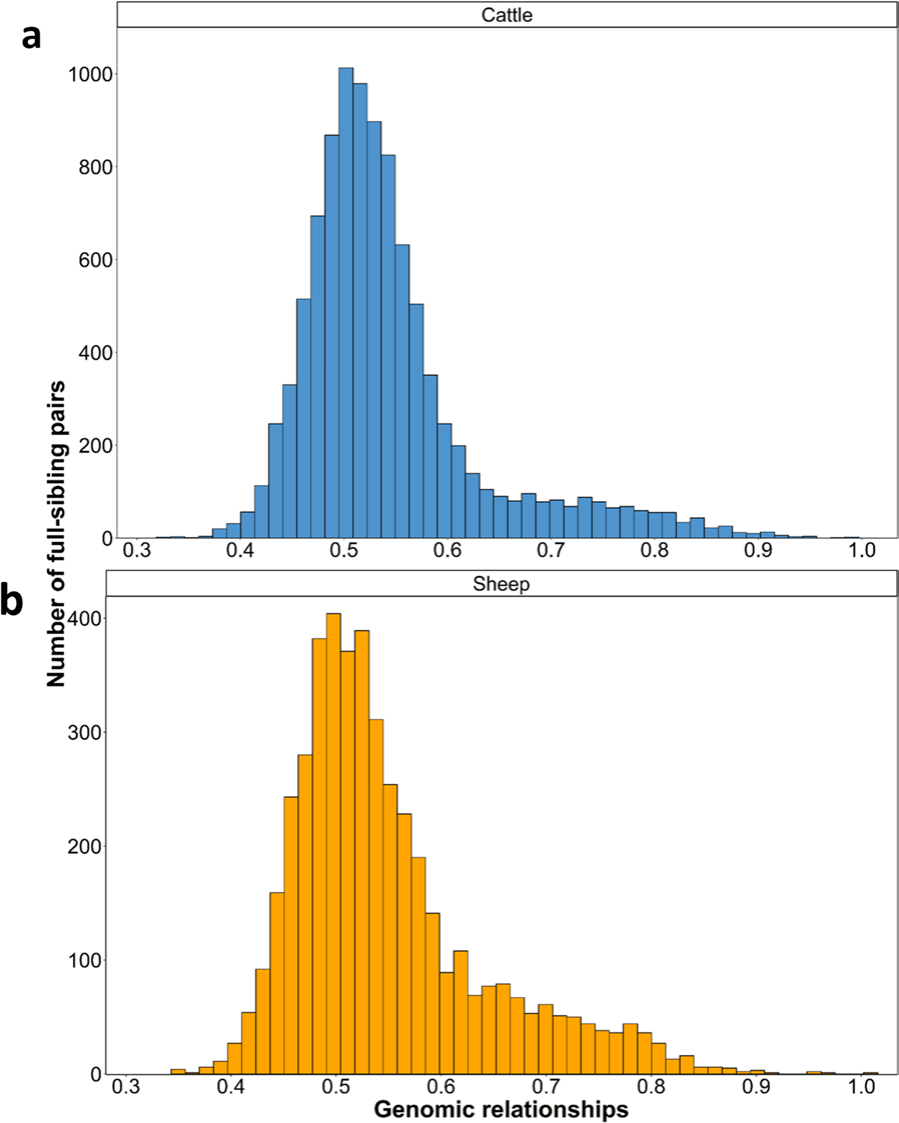
Histogram of the genomic relationships between full-sibling pairs for **a** cattle and **b** sheep

A histogram of the raw estimated genomic relationships between the full-sibling pairs of cattle and sheep is in Fig. 1. Both plots were right skewed, with a mean genomic relationship between full-siblings of 0.549 in the sheep population and 0.545 in the cattle population. For both populations, the mean genomic relationship between full-siblings was greater than 0.5 (P < 0.05). Furthermore, the standard deviation in the raw genomic relationships between full-siblings was 0.092 units in the sheep population and 0.093 units in the cattle population.

The linear regression model, which regressed each full-sibling genomic relationship on sire and dam inbreeding, as well as the genomic relationship between the parents, accounted for 84.1% of the variance in the genomic relationship between full-siblings in the sheep dataset and 80.2% of the variance in the genomic relationship between full-siblings in the cattle dataset. The genomic relationship between parents and their respective genomic inbreeding coefficients were all associated (P < 0.05) with the genomic relationships between full-siblings (Table 1). Therefore, parental inbreeding was associated with the genomic relationship between full-siblings, even after accounting for the genomic relationship between the sire and dam. The intercept and regression coefficients for each of the model terms were very similar for the cattle and sheep datasets (Table 1). Based on the regression coefficients, a 10-percentage unit increase in either the genomic relationship between the parents, sire inbreeding coefficient, or dam inbreeding coefficient would be expected to translate to an increase of 0.050, 0.024 and 0.025, respectively, in the genomic relationship between full-sibling cattle. Based on the regression coefficients, such increases would also be expected to translate to 0.050, 0.026 and 0.025, respectively, in the genomic relationship between full-sibling sheep. The intercept term, which corresponded to the mean genomic relationship between full-siblings, after accounting for the genomic relationship between the parents and the genomic inbreeding coefficients of both parents, for both datasets was approximately 0.5 (Table 1). The mean genomic relationship between parent-progeny pairs, after adjusting for parental inbreeding and the genomic relationship between the parents, was 0.499 in the sheep population with a standard deviation of 0.007 and was 0.499 in the cattle population with a standard deviation of 0.010. Furthermore, the standard deviation of the difference between pairs of full-siblings in their estimated genomic inbreeding was 0.018 in cattle and 0.011 in sheep.

**Table 1:** Estimated model parameters (standard error in parentheses) from a linear regression model (Eq. 2) regressing the genomic relationship between full-siblings on the genomic relationship between the sire and dam (α), the inbreeding coefficient of the sire ($\upbeta )$, and the inbreeding coefficient of the dam ($\upgamma )$

	Intercept	α	$\upbeta$	$\upgamma$
Cattle	0.500 (0.001)	0.501 (0.001)	0.244 (0.007)	0.249 (0.007)
Sheep	0.499 (0.001)	0.495 (0.006)	0.258 (0.009)	0.254 (0.011)

**Fig. 2:**
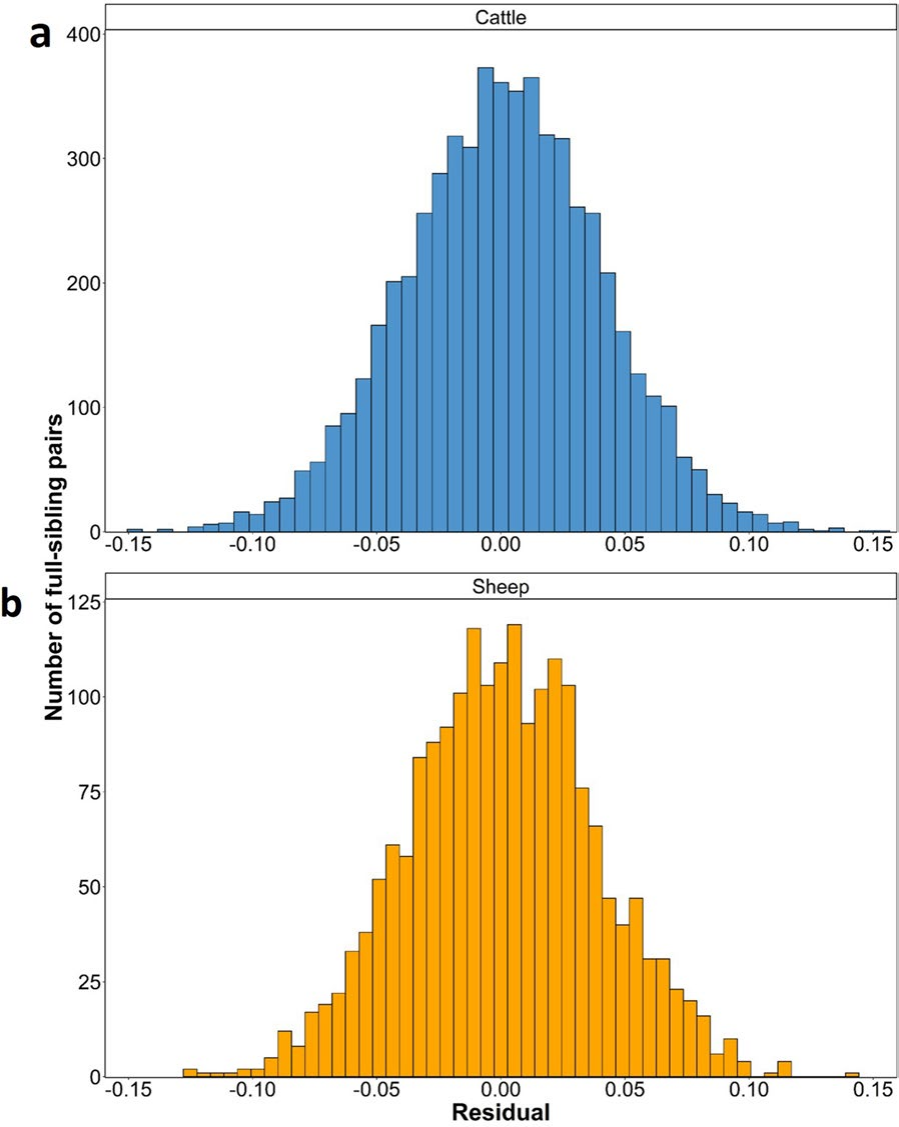
Histogram of the residual values from linear regression of the genetic relationship between full-siblings on the genomic relationship between the sire and dam, the inbreeding coefficient of the sire, and the inbreeding coefficient of the dam for the **a** cattle and **b** sheep populations

**Fig. 3:**
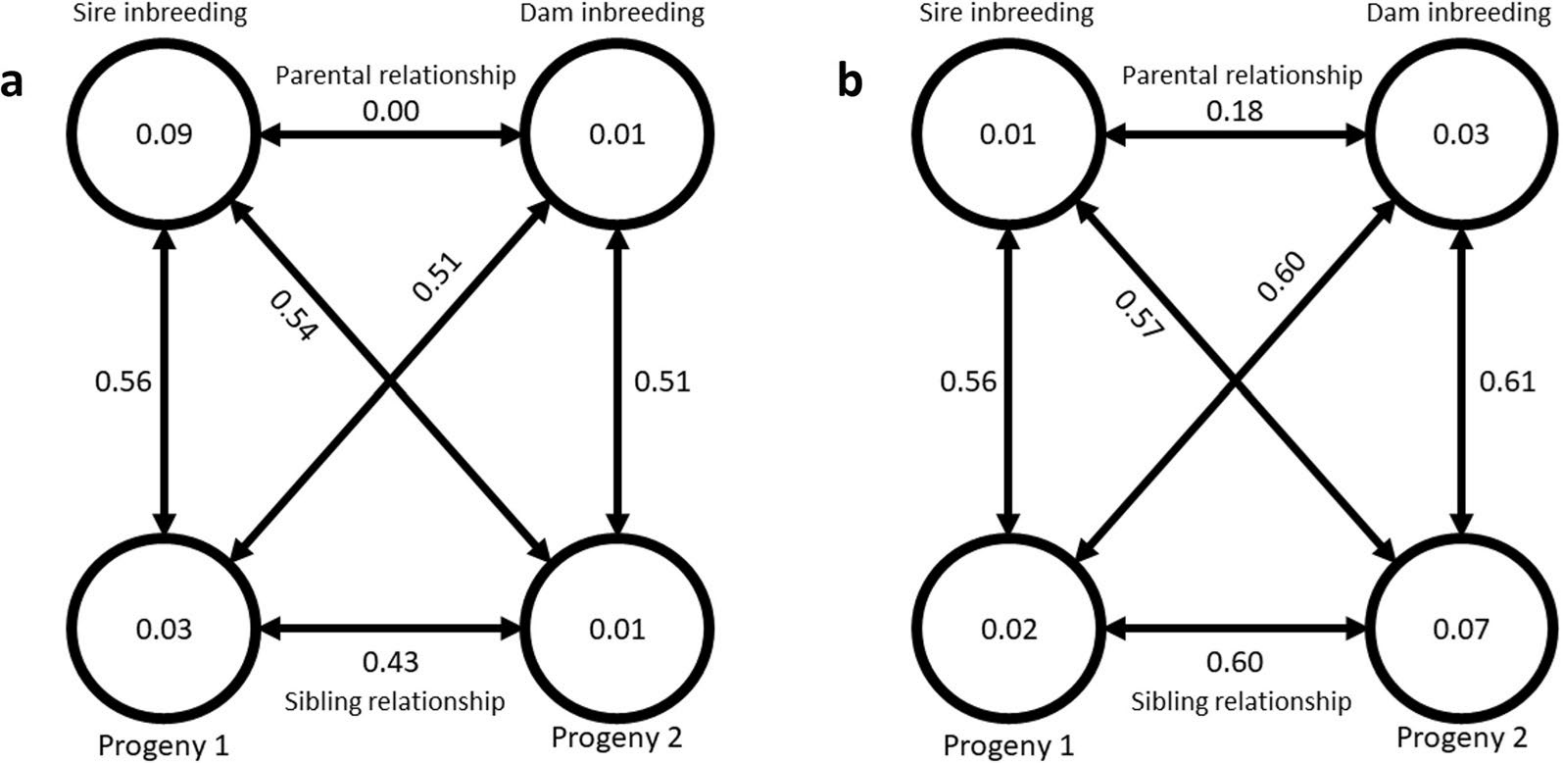
Example of two full-sibling pairs from the sheep dataset differing in their genomic relationship with their sibling [i.e., 0.43 (**a**) and 0.60 (**b**)]. The number along each double-headed arrow represents the genomic relationship between each pair of animals, while the number in each circle represents the genomic inbreeding coefficient of each animal

Histograms of the residual values from the linear regression model that regressed each full-sibling genomic relationship on sire and dam inbreeding, as well as the genomic relationship between the parents for the sheep and cattle populations are presented in Fig. 2. For both the cattle and sheep populations, the histograms of the residual values were normally distributed (Fig. 2); a QQ plot of the residual values is in Additional file 1: Figure S1 for both the cattle and sheep populations. The standard deviation of the residuals was similar in both populations, with a standard deviation of 0.037 units for the sheep population and 0.040 units for the cattle population. Similarly, using Visscher’s equation (Eq. 3), the expected standard deviation in the proportion of the genome shared between full-siblings was 0.040 for both cattle and sheep. In addition, two real examples from the sheep population of full-sibling pairs differing in their relationship with their sibling are in Fig. 3.

In a supplementary analysis to assess the impact of allele frequencies when generating the GRM, the entire analysis was re-run for the sheep population where the allele frequency of each SNP was set to 0.5. The standard deviation of the residuals from the fitted least square regression model was 0.042 units for the sheep population when values from the unweighted GRM were used in the analyses, which means that the impact of the assumed allele frequency is minimal. The populations used in the present study were heterogenous. To determine if population structure had an impact on the results of the present study, the analysis was repeated separately for the available 146 full-sibling pairs of purebred Texel sheep; the Texel population was the largest available population of purebred sheep in the present study. There was no difference in the standard deviation of the residual from the fitted least square regression model (Eq. 2) for the purebred Texel population and the entire sheep population (P < 0.05). A plot of the top four principal components of the GRM for the sheep population is presented in Additional file 1: Figure S2.

## Discussion

In the absence of inbreeding, the expected relationship between full-siblings is 0.5 [1]. In the present study, the mean genomic relationship between full-siblings was 0.545 in the cattle population and 0.549 in the sheep population, which indicates that inbreeding was present in both populations, thereby contributing to an elevated mean genomic relationship between the full-sibling pairs. Furthermore, the skewed distribution of the raw genomic relationships between full-siblings in the cattle and sheep populations indicated that variability in the parental inbreeding levels contribute to the variance in the genomic relationships between full-siblings. In both the cattle and sheep datasets, several pairs of full-siblings were highly related (Fig. 1), but they were not monozygotic twins and their genetic similarity was due to inbreeding. After accounting for both the genomic relationship between parents and parental inbreeding, the residuals of the genomic relationships between full-siblings appeared to be normal, with the mean relationship between full-siblings being approximately 0.5 in both populations (Table 1); this is in agreement with the expected relationship between out-cross full-siblings [1]. Also presented in Table 1 are the regression coefficients for the association between the genomic relationship between full-sibling pairs with the genomic relationship between the parents, sire inbreeding, and dam inbreeding. The expected regression coefficient for the parental relationship is 0.5, and 0.25 for both sire and dam inbreeding. To illustrate this, consider the following examples: first, when both parents are genetically identical and non-inbred, the expected proportion of the shared genome between siblings and between parents are both 1, hence Eq. (2) can be written as:

$$1 = 0.5 + 1\upalpha + 0\upbeta + 0\upgamma .$$

Therefore, the expected regression coefficient for the parental relationship (${\upalpha }$) is 0.5.

Second, consider unrelated parents where the sire is 100% inbred and the dam is not inbred; in this case, full-siblings will share the same genome from the sire and are expected to share half of the maternal half of their genome (i.e., 25% of the maternal genome is expected to be shared between full-siblings). In such a situation, Eq. (2) can be written as:

$$0.75 = 0.5 + 0\upalpha + 1\upbeta + 0\upgamma .$$

Hence, the expected regression coefficient for sire inbreeding ($\upbeta$) is 0.25. Similarly, the expected regression coefficient for dam inbreeding ($\upgamma$) is also 0.25. In this study, the actual regression coefficients for the genetic relationship between parents, sire inbreeding, and dam inbreeding (Table 1) conform well with the expected regression coefficients for each of these model features. What these regression coefficients also signify is that each unit increase in genomic relationship between parents will contribute a greater increase in genomic relationship between full-siblings, than an equivalent unit increase in the inbreeding coefficient of either the sire or the dam.

The residuals from the model also mean that, in the absence of inbreeding and parental co-ancestry, the standard deviation in the genomic relationships between full-siblings due to Mendelian sampling was similar in the two species of interest, namely cattle and sheep. In addition, the standard deviations estimated separately in cattle and sheep are in agreement with similar studies conducted in chickens [6] and humans [5], as well as the theoretical expectation of the standard deviation in the actual genetic relationship between full-siblings in cattle and sheep. Together these studies highlight that the genomic estimate of relationships has the potential to be more accurate than pedigree-based estimates of genetic relationships and therefore, could lead to a faster rate of genetic improvement if implemented in a selective breeding program. However, other factors will also impact the potential benefits from genotyping, such as the heritability of the trait, the accuracy of phenotype and environmental measurements, the accuracy and depth of pedigree records, the economic value of the trait, and the opportunity cost of genotyping. One of the factors which can impact mate selection in selective breeding programs is the genetic relationship between the prospective sire and dam. The results of the present study suggest that the genome-based estimate of relationships is expected to be more accurate than a pedigree-based estimate, which may facilitate favourable mate pair selection that would otherwise have been excluded due to the apparent risk of inbreeding in the progeny as suggested by just the pedigree information. Conversely, the use of genomic relationship estimates may exclude mate pairs which are more closely related than indicated by their respective pedigree information and thereby reduce the rate of accumulation of inbreeding within a population. Having a more accurate estimate of the expected inbreeding for different candidate mate pairs is important in cattle mating advice decision support systems that penalize each pairwise parental mating option on expected inbreeding [14].

In spite of the fact that inbreeding can be reversed after a single generation of out-crossing [15], our results confirm for the analysed populations that offspring born to unrelated, but inbred parents will tend to be more closely related to one another. Therefore, those offspring will have less genetic diversity than offspring from unrelated non-inbred parents, which is consistent with the theoretical expectations as outlined by Hill and Weir [16]. Nonetheless, although both siblings have the same parents, estimated genomic inbreeding coefficients differ between full-siblings, which is an outcome of independent assortment of haplotypes to sister chromatids during gamete formation. The results of this analysis have important implications for breeding and management strategies for cattle and sheep where inbreeding can be incidental due to the low effective population sizes of cattle and sheep [17, 18]. This can also be the case for species such as chickens and pigs, where it is common to perform intense selection within purebred lines of animals for the purposes of outcrossing [19, 20]. Employing breeding strategies that promote genetic diversity can be important for domestic livestock because populations with lower genetic diversity have a reduced capacity to respond to selection [21]. Furthermore, reduced genetic diversity can also increase the likelihood of inbreeding in future generations [21]. Therefore, breeders who wish to promote genetic diversity within their populations should consider not only the expected inbreeding coefficient of progeny, but also the inbreeding coefficient of the sire and dam, even when the sire and dam in question are unrelated.

## Conclusions

Our results show that the standard deviation in genomic relationships between full-sibling cattle and sheep was similar, with the standard deviation being 0.040 and 0.037 units, respectively, in cattle and sheep, which is close to the theoretical expectation. These standard deviations were adjusted for sire and dam inbreeding, as well as for the genomic relationship between the parents, all of which were associated with the genomic relationships between their full-sibling progeny. In addition, the intercept of the model was 0.50 in both the cattle and sheep datasets, which means that, as expected, in the absence of inbreeding, full-siblings, on average, share 50% of their segregating genome. Knowledge of the variation in genomic relationships among individuals relative to the expectation may be useful when evaluating the potential opportunity costs of using genomic information or pedigree information in genetic evaluations of domestic livestock.

## Supplementary Information

**Additional file 1: Figure S1.** Quantile–quantile plots of the model residuals from the models used to adjust the genomic relationships between full-sibling cattle (a) and sheep (b) pairs. **Figure S2.** Scatter plot of the top four principal components of the genomic relationship matrix (GRM) for the full-sibling sheep population. Crossbred sheep are represented by brown dots, Belclare are in blue, Suffolk are in yellow, Texel are in purple, and Vendeen are in green.

## Data Availability

Individual genotype and phenotype data used in this study are managed by third parties, namely the Irish Cattle Breeding Federation and Sheep Ireland. Reasonable requests for the cattle data can be made to the Irish Cattle Breeding Federation (email: query@icbf.com; website: https://www.icbf.com/), while for the sheep data such requests can be directed to Sheep Ireland (email: query@sheep.ie; website: https://www.sheep.ie/).
