# Supplementary material for: Variation in the proportion of the segregating genome shared between full-sibling cattle and sheep
Source: Genet Sel Evol. 2023 Apr 18;55:27. doi: 10.1186/s12711-023-00802-5 (PMC10111659; doi:10.1186/s12711-023-00802-5)
Supplement: Supplementary file 1 — Additional file 1: Figure S1. Quantile–quantile plots of the model residuals from the models used to adjust the genomic relationships between full-sibling cattle (a) and sheep (b) pairs. Figure S2. Scatter plot of the top four principal components of the genomic relationship matrix (GRM) for the full-sibling sheep population. Crossbred sheep are represented by brown dots, Belclare are in blue, Suffolk are in yellow, Texel are in purple, and Vendeen are in green. [file 12711_2023_802_MOESM1_ESM.docx]

**Supplementary material**


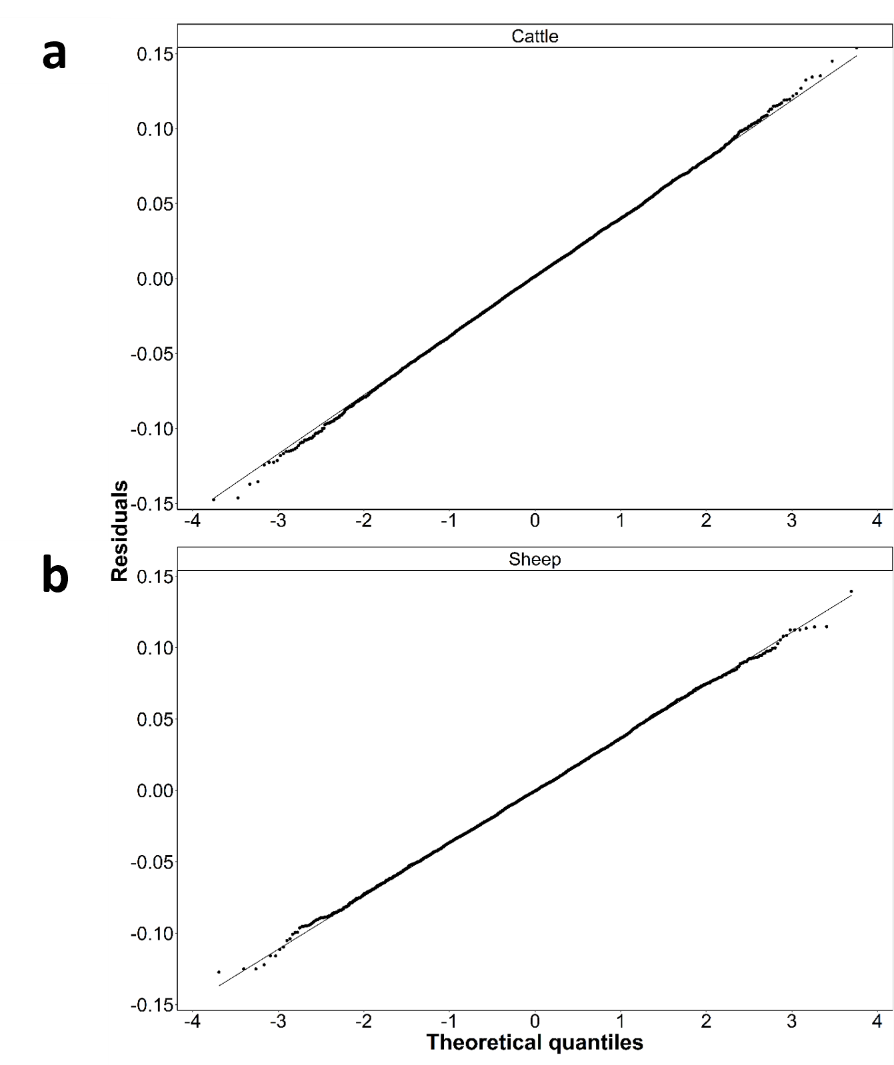


**Supplementary Figure S1** Quantile-quantile plots of the model residuals from the models used to adjust the genomic relationships between full-sibling cattle (a) and sheep (b) pairs.


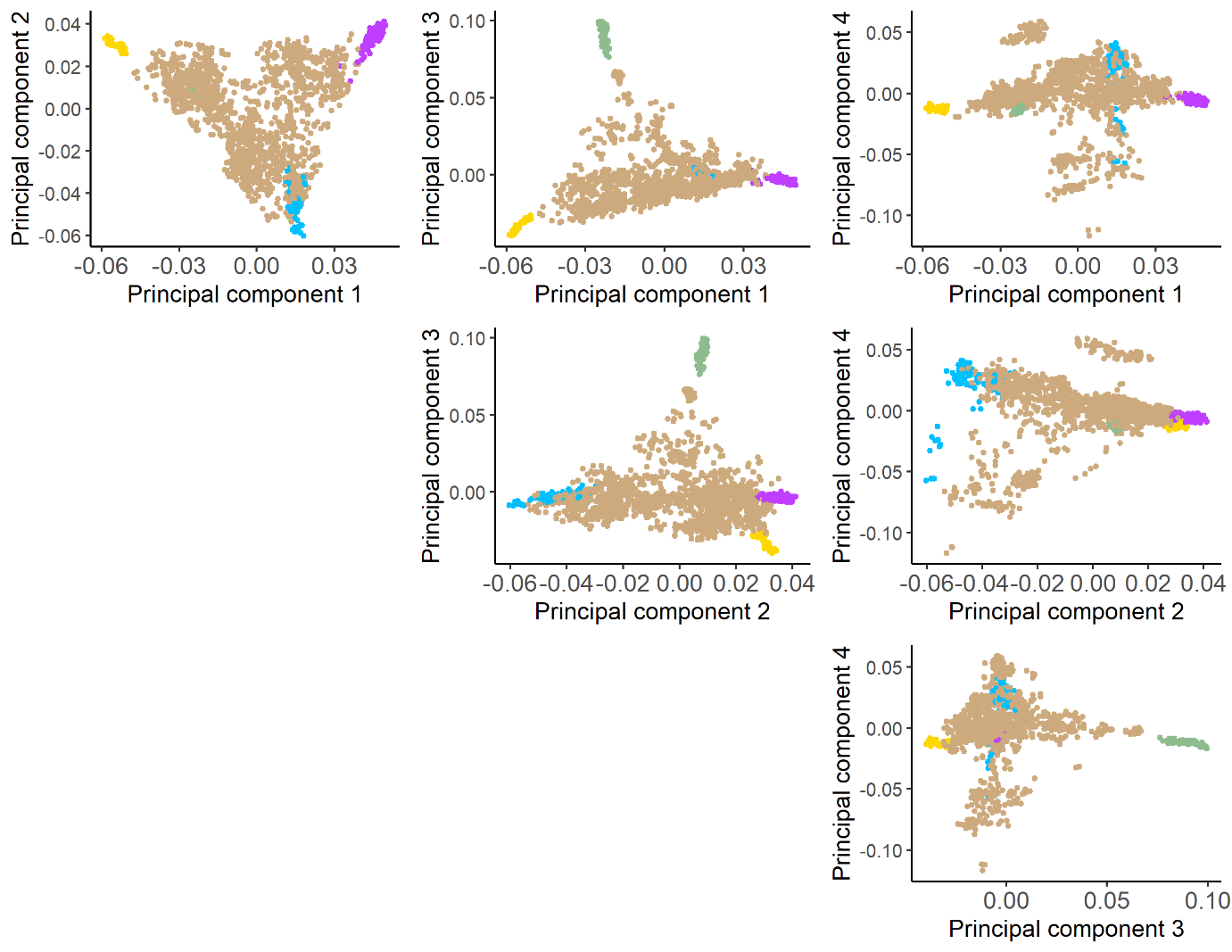


**Figure S2** Scatter plot of the top four principal components of the genomic relationship matrix (GRM) for the full-sibling sheep population. Crossbred sheep are represented by brown dots, Belclare are blue, Suffolk are yellow, Texel are purple, and Vendeen are green.
